# S3PM: Entropy-Regularized Path Planning for Autonomous Mobile Robots in Dense 3D Point Clouds of Unstructured Environments

**DOI:** 10.3390/s26020731

**Published:** 2026-01-21

**Authors:** Artem Sazonov, Oleksii Kuchkin, Irina Cherepanska, Arūnas Lipnickas

**Affiliations:** 1 Automation Hardware and Software Department, National Technical University of Ukraine “Igor Sikorsky Kyiv Polytechnic Institute”, 37, Prospect Beresteiskyi, 03056 Kyiv, Ukraine; a.kuchkin@lll.kpi.ua; 2 Department of Automation and Non-Destructive Testing Systems, National Technical University of Ukraine “Igor Sikorsky Kyiv Polytechnic Institute”, 37, Prospect Beresteiskyi, 03056 Kyiv, Ukraine; cherepanskairina@gmail.com; 3 Department of Automation, Kaunas University of Technology, 51367 Kaunas, Lithuania

**Keywords:** path planning, robot control, mobile robots, entropy, point cloud, unstructured environment, computer vision

## Abstract

Autonomous navigation in cluttered and dynamic industrial environments remains a major challenge for mobile robots. Traditional occupancy-grid and geometric planning approaches often struggle in such unstructured settings due to partial observability, sensor noise, and the frequent presence of moving agents (machinery, vehicles, humans). These limitations seriously undermine long-term reliability and safety compliance—both essential for Industry 4.0 applications. This paper introduces S3PM, a lightweight entropy-regularized framework for simultaneous mapping and path planning that operates directly on dense 3D point clouds. Its key innovation is a dynamics-aware entropy field that fuses per-voxel occupancy probabilities with motion cues derived from residual optical flow. Each voxel is assigned a risk-weighted entropy score that accounts for both geometric uncertainty and predicted object dynamics. This representation enables (i) robust differentiation between reliable free space and ambiguous/hazardous regions, (ii) proactive collision avoidance, and (iii) real-time trajectory replanning. The resulting multi-objective cost function effectively balances path length, smoothness, safety margins, and expected information gain, while maintaining high computational efficiency through voxel hashing and incremental distance transforms. Extensive experiments in both real-world and simulated settings, conducted on a Raspberry Pi 5 (with and without the Hailo-8 NPU), show that S3PM achieves 18–27% higher IoU in static/dynamic segmentation, 0.94–0.97 AUC in motion detection, and 30–45% fewer collisions compared to OctoMap + RRT* and standard probabilistic baselines. The full pipeline runs at 12–15 Hz on the bare Pi 5 and 25–30 Hz with NPU acceleration, making S3PM highly suitable for deployment on resource-constrained embedded platforms.

## 1. Introduction

Autonomous navigation in unstructured industrial environments remains one of the most critical challenges in mobile robotics. Unlike highly structured domains such as warehouses or assembly lines, industrial facilities exhibit irregular layouts, cluttered workspaces, frequent equipment reconfiguration, and the constant presence of moving agents—including machinery, automated guided vehicles (AGVs), unmanned aerial vehicles (UAVs), and human operators. These factors introduce substantial uncertainty and unpredictability, significantly complicating reliable localization, robust mapping, and safe path planning compared to controlled settings [1,2,3,4].

The demand for flexible automation driven by Industry 4.0 has further intensified the need for robotic systems capable of seamless adaptation to changing environments while operating safely alongside humans. Failure to achieve robust navigation not only compromises operational efficiency and increases downtime but also poses serious safety risks, including potential collisions with personnel or valuable equipment. Consequently, the development of real-time methods that can effectively handle both static clutter and dynamic uncertainty is essential for the large-scale deployment of autonomous mobile robots in industrial applications [5,6].

Classical mapping and motion-planning approaches—such as occupancy-grid mapping combined with graph-search algorithms (A*, D*) [7,8] or sampling-based planners (RRT*, PRM) [9,10,11,12]—have been widely successful in structured or semi-structured environments. However, these methods rely on rigid environmental assumptions and struggle in cluttered, partially observable, or highly dynamic 3D spaces [13,14,15]. Probabilistic volumetric frameworks such as OctoMap [2] have improved scalability and uncertainty modeling; nevertheless, they typically do not incorporate explicit motion cues and remain computationally expensive when updating large-scale maps in real time.

Furthermore, while state-of-the-art local planners such as the Timed Elastic Band (TEB) [16,17] and Model Predictive Path Integral (MPPI) control [18,19] excel at dynamic obstacle avoidance, they impose substantial computational demands that are often prohibitive for low-power embedded edge devices lacking dedicated GPUs. Simpler reactive methods, such as the Dynamic Window Approach (DWA) [20], are computationally efficient but frequently fail in complex environments due to clutter-induced local minima. More recent learning-based approaches [21,22,23] have demonstrated impressive performance in simulation; however, they typically require large training datasets, exhibit limited generalization beyond the training distribution, and are rarely suitable for resource-constrained embedded platforms because of latency and memory limitations.

In this work, we propose S3PM, an entropy-regularized framework for simultaneous mapping and path planning that operates directly on dense 3D point clouds. The core innovation is a dynamic-aware entropy field that fuses voxel-wise occupancy probabilities with motion evidence derived from residual optical flow. This representation enables the system to distinguish reliably navigable space from ambiguous or high-risk regions, supports proactive collision prediction, and facilitates real-time trajectory correction. Importantly, the method is designed with embedded deployment in mind, achieving real-time performance on low-power platforms such as the Raspberry Pi 5.

Through extensive evaluation in both simulated and real-world industrial scenarios, we demonstrate that S3PM substantially improves navigation robustness and safety while maintaining computational efficiency. The proposed approach establishes a foundation for entropy-driven navigation strategies that generalize beyond industrial settings and advance the broader field of autonomous mobile robotics in unstructured environments.

## 2. Materials and Methods

### 2.1. Related Work

Research on mobile robot navigation has produced a wide variety of approaches, ranging from classical geometric planning methods [24] to modern learning-based techniques [21,22,23]. While each of these paradigms has demonstrated progress in specific domains, significant limitations remain when transferring them to dense, unstructured 3D indoor environments, where both static and dynamic uncertainties are prevalent.

One of the most established families of approaches is based on occupancy-grid mapping and graph-search algorithms such as A*, D*, and their variants [7,8]. These methods are computationally efficient and provide deterministic guarantees in structured settings, such as warehouses or predefined floor layouts. However, their reliance on discretized 2D projections or simplified geometric assumptions severely limits their performance in cluttered 3D spaces. The absence of a principled mechanism for quantifying uncertainty further makes these planners brittle when faced with noisy or ambiguous sensor data (e.g., from depth cameras or LiDAR). As noted by several authors [14,15,25,26], despite decades of refinement, classical search-based methods often fail to generalize to unstructured and dynamic conditions without extensive manual tuning.

Another major group of approaches focuses on sampling-based motion planning, including algorithms such as Rapidly Exploring Random Trees (RRT) [27,28,29] and Probabilistic Roadmaps (PRM) [30]. These planners are well suited to high-dimensional search spaces and have been widely applied in both terrestrial (AGVs) and aerial robotics (UAVs). Nonetheless, they are highly sensitive to the underlying map representation: when applied to raw or voxelized point clouds, they often require costly preprocessing steps (e.g., meshing or smoothing) to avoid spurious connections. Moreover, sampling-based planners typically treat the environment as static during each planning cycle, which limits their applicability in scenarios involving moving agents or reconfigurable obstacles [21].

In the domain of dynamic collision avoidance, local trajectory optimization methods have become a standard solution. The Dynamic Window Approach (DWA) [20] offers a lightweight reactive strategy but suffers from a short prediction horizon, frequently leading to dead ends in complex industrial layouts. To mitigate this limitation, optimization-based methods such as the Timed Elastic Band (TEB) planner [17] deform trajectories to avoid obstacles while respecting kinematic constraints. However, TEB becomes computationally expensive in dense 3D environments and is particularly sensitive to noisy point cloud data. Model Predictive Path Integral (MPPI) control [19] demonstrates state-of-the-art performance in highly dynamic settings by sampling thousands of candidate trajectories. Despite its robustness, MPPI typically requires massive parallelization on powerful GPUs to achieve real-time performance [18,19], rendering it unsuitable for cost-effective, energy-constrained embedded platforms such as the Raspberry Pi without substantial hardware acceleration.

Besides the above-mentioned groups of algorithms, the Probabilistic Occupancy Mapping techniques, particularly those derived from Bayesian filtering and SLAM frameworks, have been proposed to explicitly model uncertainty in perception. A prominent representative of such techniques, like OctoMap [2], attempts to maintain a volumetric belief distribution over free and occupied spaces. While powerful in outdoor robotics and aerial mapping, these methods suffer from scalability and responsiveness issues in dense indoor settings, as updating a large 3D map in real time is computationally expensive. Furthermore, these techniques generally do not incorporate motion cues from optical flow or temporal dynamics, making them ill-suited for environments where static and dynamic entities must be separated [23].

More recently, learning-based methods have gained attention [26,27,28], leveraging convolutional neural networks (CNNs), graph neural networks (GNNs), or reinforcement learning to directly infer navigation strategies from sensor data. These approaches can, in principle, capture complex scene semantics and adapt to dynamic conditions. However, they remain limited by their dependence on large training datasets, difficulties in generalizing beyond the training distribution, and heavy computational requirements. As highlighted in a review [22], many deep learning planners achieve impressive simulation performance but are rarely deployed in resource-constrained embedded systems due to latency and memory bottlenecks.

In contrast to these approaches, the proposed method integrates entropy-regularized mapping with dynamic likelihood estimation, thereby unifying uncertainty modeling and motion awareness into a single probabilistic path planning method (S3PM) described below. This allows the system to remain lightweight enough for embedded execution while explicitly reasoning about ambiguous and dynamic regions of the environment, a combination not adequately addressed in prior works.

### 2.2. S3PM Explanation

The proposed method first defines the evaluation criteria and regularization terms, followed by a description of the optimization process used to determine the mobile robot’s optimal path.

#### 2.2.1. Entropy-Guided Environment Representation

**Figure 1:**
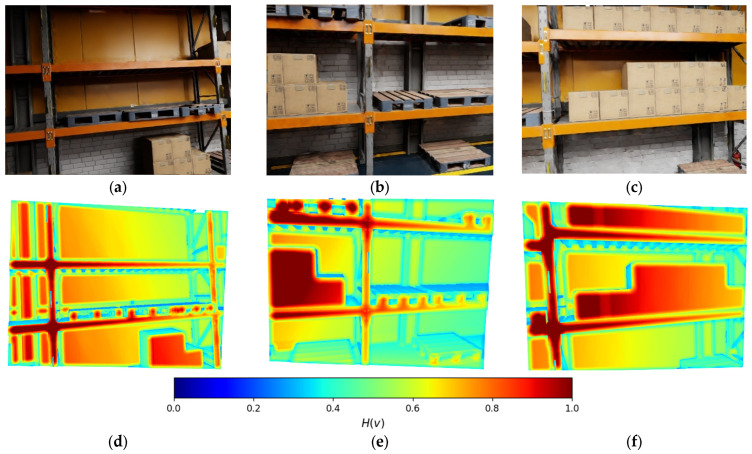
Entropy field of a point cloud (**d**–**f**) for the corresponding RGB scene representation (**a**–**c**): blue—0.0, green—0.5, red—1.0.

To enable safe and efficient path planning for mobile robotic platforms operating in unstructured indoor environments, an entropy-driven representation of the environment based on 3D point clouds is introduced (Figure 1). Rather than operating directly on raw point clouds, a voxel-based representation using spatial hashing is employed. This design choice is motivated by the need for high-frequency map updates on embedded hardware platforms. Voxelization abstracts away sub-voxel geometric detail and aggregates dense sensor measurements into discrete spatial units. Instead of performing computationally expensive nearest-neighbor searches for individual points, the probabilistic parameters of the corresponding voxels can be accessed and updated in constant time, *O*(1). As a result, the global map state and its associated entropy can be refreshed at each incoming camera frame without introducing computational bottlenecks. Each voxelized region is subsequently assigned an entropy score that quantifies the uncertainty and variability of the local geometry. The entropy *H* of a voxel *v* is defined as:

(1) $$H\left(v\right)=-\sum_{i=1}^{k}p_{i}\log\left(p_{i}\right),$$

where *p_i_* denotes the probability of the occupancy states (free, occupied, dynamic, unknown), computed according to (6). High-entropy regions correspond to ambiguous or unstable structures (e.g., cluttered areas or moving objects), whereas low-entropy regions represent either reliably free or reliably occupied space.

This entropy map enables dividing the environment into navigable and non-navigable zones. Regions with entropy below a chosen threshold are considered safe candidates for traversal, whereas high-entropy zones are treated as obstacles with high uncertainty or dynamic risk.

Furthermore, regions containing uncertain obstacles should be evaluated in conjunction with pixel-level motion estimates obtained from optical flow, as dynamic objects typically influence extended regions of the map along their direction of motion.

**Figure 2:**
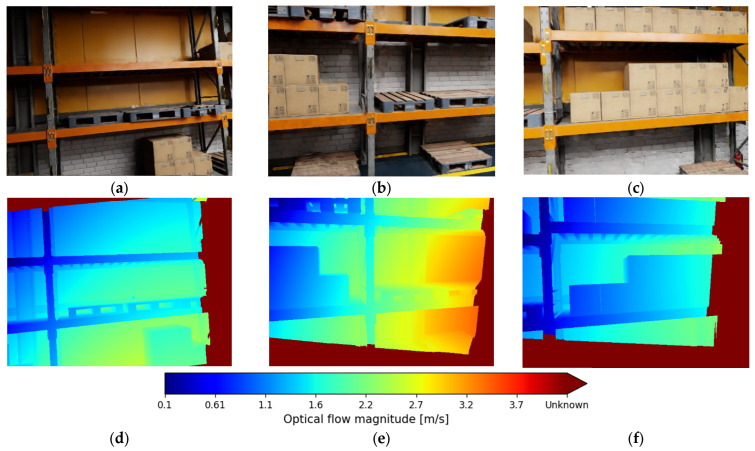
Optical flow field of a point cloud (**d**–**f**) for the corresponding RGB scene representation (**a**–**c**): blue—less than 0.1 m/s; green—2.2 m/s; red—more than 4.2 m/s (or unknown).

Thus, for each image pair ($I_{t},I_{t+\Delta }$) semi-dense optical flow f(u) is estimated at pixel u (Figure 2). Given pose change $T_{t\to t+\Delta }$ and per-pixel depth z(u), the expected flow due to static background (ego-motion only) is calculated. For a pixel u=(x,y) with normalized coordinates, the expected motion field $\overset{ˆ}{f}\left(u\right)=(\dot{u},\dot{v})$ induced purely by ego-motion is derived from the pinhole model:

(2) $$\left[\begin{array}{l} \dot{u}\\ \dot{v} \end{array}\right]=\left[\begin{array}{ccc} -1/z & 0 & x/z\\ 0 & -1/z & y/z \end{array}\right]v+\left[\begin{array}{ccc} xy & -\left(1+x^{2}\right) & y\\ \left(1+y^{2}\right) & -xy & -x \end{array}\right]\omega ,$$

where v—linear velocity and ω—angular velocity both are calculated from the VIO pipeline. Thus, the residual flow r(u) (3) is small for a static background and large where independently moving objects are present.

(3) $$r(u)=f(u)-\overset{ˆ}{f}(u).$$

The residual magnitudes are passed through a robust sigmoid to obtain dynamic likelihoods:

(4) $$l_{dyn}(u)=w_{\mathrm{vis}}\sigma \left(\frac{\|r(u)\|-\tau }{\sigma_{r}}\right),$$

where τ∈(0,255) is the threshold tied to photometric noise, $\sigma_{r}=127$—a scale parameter, σ− sigmoid function $w_{\mathrm{vis}}$ —weights low-texture regions. To mitigate errors in texture-less regions where optical flow is unreliable, the weight $w_{\mathrm{vis}}$ in (3) is derived from the local image gradient magnitude |∇I|. If $|\nabla I|<\delta_{\mathrm{texture}},w_{\mathrm{vis}}\to 0$, preventing false dynamic positives on plain walls. Furthermore, localization errors typically cause global spikes in the residual flow. These are filtered by enforcing consistency checks on residual flow directions within local pixel neighborhoods, thereby rejecting random noise induced by drift. Once a confident dynamic likelihood is obtained at the pixel level, this evidence is propagated into the volumetric map using the following ray-based update rules:

- Along the ray until the first confident surface, the free-space evidence is added;

- At the surface voxel, the evidence is split between occupied vs. dynamic by using $l_{dyn}$ (e.g., add $l_{dyn}$ to $n^{dyn}$, $1-l_{dyn}$ to $n^{\mathrm{stat}\;}$);

- Voxels never intersected by valid rays retain unknown mass.

To avoid jitters, temporal consistency is applied with exponential smoothing of dynamic likelihoods for object tracks (e.g., person/forklift) using short-term association in image space (optical flow) or 3D (nearest-neighbor on centroids):

(5) $${\tilde{l}}_{dyn,t}=\beta {\tilde{l}}_{dyn,t-1}+(1-\beta )l_{dyn,t,}$$

where β∈[0,1]—smoothness parameter; ${\tilde{l}}_{dyn,t-1}$—previous dynamic likelihood; ${\tilde{l}}_{dyn,t}$—current dynamic likelihood.

#### 2.2.2. Probabilistic Updates and the Dynamic-Aware Entropy Field

The approach employs per-voxel Dirichlet-multinomial Bayesian updates to ensure robust probabilities under sparse observations. The state of a voxel v is modeled by a categorical distribution over S={free, stat, dyn, unk} with parameters derived from accumulated pseudo-counts $\alpha_{t}(v)$. Unlike standard occupancy grids that update log-odds directly, the sensory evidence is explicitly mapped into count increments $\Delta n^{s}$. Voxel updates occur by unprojecting rays from valid pixels (with computable depth and optical flow) from the camera as follows:

1. All voxels v along the ray path (excluding the endpoint) receive evidence assigned to the free state:

(6) $$\Delta n^{\mathrm{free}}(v)=w_{\mathrm{vis}},\Delta n^{\frac{\mathrm{stat}}{dyn}}(v)=0.$$

2. At the endpoint voxel $v_{\mathrm{hit}}$, the evidence is split between static and dynamic occupancy based on the dynamic likelihood derived in (5):

(7) $$\Delta n^{dyn}\left(v_{hit}\right)={\tilde{l}}_{\mathrm{dyn}}\left(u\right),$$

(8) $$\Delta n^{stat}\left(v_{hit}\right)=\left(1-{\tilde{l}}_{dyn}(u)\right).$$

3. Spatial unpredictability of moving agents (e.g., motion blur or rapid position changes) prompts strict increases in unknown evidence within the immediate neighborhood $N\left(v_{hit}\right)$ of voxels identified as dynamic. If ${\tilde{l}}_{dyn}\left(u\right)>0.5$, then for all neighbors $v^{\prime }\in N\left(v_{hit}\right)$

(9) $$\Delta n^{unk}\left(v^{\prime }\right)=\frac{1}{\left\|v^{\prime }-v_{hit}\right\|}\cdot {\tilde{l}}_{\mathrm{dyn}}\left(u\right).$$

This crucial step ensures that the entropy field spikes not only at the moving object but also in its vicinity, forcing the planner to maintain a safety buffer due to high epistemic uncertainty.

These instantaneous increments are used to update the Dirichlet parameters $\alpha_{t}(v)=\left[\alpha_{t}^{free},\alpha_{t}^{stat},\alpha_{t}^{dyn},\alpha_{t}^{unk}\right]$:

(10) $$\alpha_{t+1}^{s}\left(v\right)=\lambda \alpha_{t}^{s}\left(v\right)+\Delta n^{s}\left(v\right),s\in S,$$

where λ∈(0,1]—forgetting factor (smaller λ lets the map adapt quicker to layout changes). Probabilities follow the posterior mean:

(11) $$p_{t+1}^{s}(v)=\frac{\alpha_{t+1}^{s}(v)}{\sum_{s^{\prime }\in S}\alpha_{t+1}^{s^{\prime }}(v)}.$$

**Figure 3:**
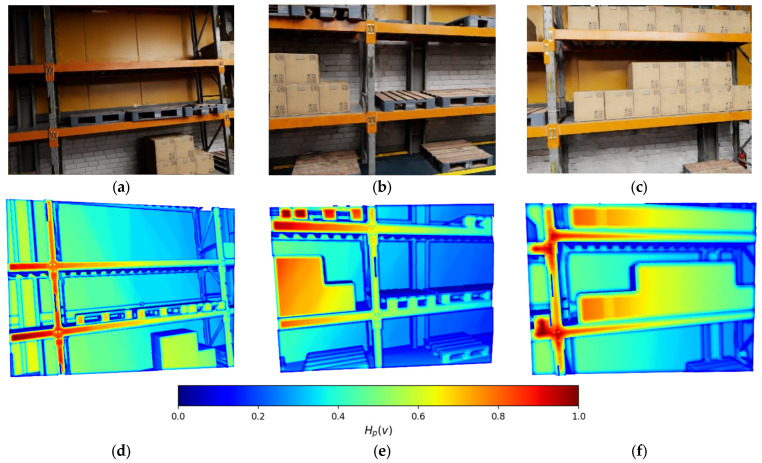
Dynamic-aware entropy field of a point cloud (**d**–**f**) for the corresponding RGB scene representation (**a**–**c**): blue—0.0; green—0.5; red—1.0.

We define the dynamic-aware entropy per voxel (Figure 3):

(12) $$H\left(v\right)=-\sum_{s\in S}p^{s}\left(v\right)\log p^{s}\left(v\right).$$

While conventional planners often use a linear combination of static and dynamic occupancy probabilities $p^{\mathrm{stat}\;}+p^{dyn}$ as a cost term, this formulation fails to distinguish between known obstacles and epistemic uncertainty. For instance, a voxel with p=[0.5,0.5] (high ambiguity) and a voxel with p=[0.9,0.1] (representing high certainty) may yield similar linear costs in standard risk maps. In contrast, the entropy-based formulation in (8) assigns a substantially higher penalty to the ambiguous case. As a result, the planner is explicitly encouraged to avoid regions in which the perception system exhibits high uncertainty (e.g., irregular optical flow patterns or sparse sensor data), rather than merely avoiding clearly detected static obstacles such as walls.

Dynamic occupancy introduces additional operational risk; therefore, risk-weighted entropy is incorporated into the planning objective:

(13) $$H_{\rho }\left(v\right)=-\sum_{s\in S}\rho_{s}p^{s}\left(v\right)\log p^{s}\left(v\right),\rho_{dyn}>\rho_{stat}\geq \rho_{free},$$

where ρ scales the penalty of uncertainty according to downstream safety.

#### 2.2.3. Navigability Classification from Entropy and Risk

Occupancy probability p(v) and $H_{\rho }(v)$ yield navigability score $C_{nav}(v)$ for the planner:

(14) $$C_{\mathrm{nav}}(v)=w_{\mathrm{occ}}\left(p^{\mathrm{stat}}(v)+p^{\mathrm{dyn}}(v)\right)+w_{\mathrm{ent}}H_{\rho }(v)+w_{\mathrm{clr}}\Phi_{\mathrm{clear}}(v),$$

where $\Phi_{\mathrm{clear}}(v)$—penalty function for poor clearance from likely occupied voxels. By explicitly separating the occupancy probability term $w_{\mathrm{occ}}$ from the entropy term $w_{\mathrm{ent}}$, the cost function (18) enables decision behavior that standard probability-weighted maps cannot achieve. Unlike conventional risk maps, where a probability of p=0.5 typically yields a linear cost equivalent to a semi-traversable terrain (often leading to risky shortcuts through ambiguous areas), the entropy term $H\left(v\right)$ acts as a non-linear barrier against epistemic uncertainty. This formulation results in distinct path selection behavior: the planner effectively penalizes unobserved regions as heavily as known obstacles, forcing the robot to select longer paths through verified free space rather than shorter paths through ambiguous data. Such differentiation enables the robot to be aggressive near known obstacles (low H, high p) but conservative near ambiguous data (high H), or vice versa. Thresholds define classes:

(15) $$\tau_{\mathrm{free}}\leq C_{\mathrm{nav}}(v)<\tau_{\mathrm{caut}}\Rightarrow \begin{array}{ll} C_{\mathrm{nav}}\left(v\right)<\tau_{\mathrm{free}} & \Rightarrow \mathrm{Free}\\ C_{\mathrm{nav}}\left(v\right)\geq \tau_{\mathrm{caut}} & \Rightarrow \mathrm{Blocked} \end{array}.$$

By design, regions with dynamic ambiguity (high $p^{dyn}$ or high $H_{\rho }$) shift to Caution/Blocked even if instantaneous geometry looks permissive; this is critical near human–robot interaction zones.

#### 2.2.4. Incremental Map Expansion and Uncertainty Reduction

Industrial deployments require growing the known map while maintaining production throughput. Expansion is formalized via frontiers and information gain. A voxel is classified as a frontier if it is adjacent to unknown space:

(16) $$F=\left\{v|p^{unk}(v)>\eta_{unk}\wedge \exists v^{\prime }\in N(v):p^{unk}\left(v^{\prime }\right)\leq \eta_{unk}\right\}.$$

The Expected Information Gain (*EIG*) for visiting neighborhood N along a path segment π is approximated by

(17) $$EIG\left(\pi \right)=\sum_{v\in N\left(\pi \right)}\kappa \left(v\right)H\left(v\right),$$

where κ(v)∈[0,1]—visibility factor capturing whether the robot’s camera is likely to observe v given line-of-sight and view geometry (efficiently approximated using a ray bundle within a local frustum). Because moving objects can increase uncertainty over time, the Dirichlet update with forgetting (10) ensures revisits consolidate evidence: stable structures see $p^{\mathrm{stat}}↑\Rightarrow H↓$, while persistently dynamic regions keep H high and can be deliberately avoided in production routes.

We embed exploration into planning via an information bonus that competes with entropy penalties (13). In “mapping mode”, *EIG* can dominate to promote discovery; in “production mode”, only marginal frontier bonuses remain, favoring low-risk, well-known corridors.

#### 2.2.5. Multi-Criteria Path Optimization with Entropy Regularization

Given a start $x_{0}$ and goal $x_{g}$, the optimal path $\pi ={\left\{x_{t}\right\}}_{t=0}^{T}$ is searched on a 3D grid using the cost function (13). The composite objective balances travel efficiency, smoothness, safety under uncertainty, and map expansion.

(18) $$J\left(\pi \right)=\sum_{t=0}^{T}\left(w_{d}\left\|x_{t+1}-x_{t}\right\|+w_{\kappa }\kappa_{\mathrm{curv}}\left(x_{t}\right)+w_{\mathrm{nav}}C_{\mathrm{nav}}\left(v\left(x_{t}\right)\right)-w_{\mathrm{eig}}{EIG}_{\mathrm{local}}\left(x_{t}\right)\right),$$

where $\kappa_{\mathrm{curv}}$ penalizes curvature (task-dependent smoothness), $C_{\mathrm{nav}}$ is from (14), and ${EIG}_{\mathrm{local}}$ is a local approximation of (17) computed over a sliding window around $x_{t}$. Tuning:

The weights of the individual components in the cost function (18) are determined empirically based on the target processing pipeline, using sensitivity analysis on a simulated dataset. Table 1 summarizes the key characteristics and illustrates the effect of different weight configurations on overall pipeline performance.

The optimization problem J utilizes graph search via a sampling-based planner (e.g., RRT* with entropy-biased sampling density $p_{\mathrm{sample}}(x)\propto e^{-\gamma C_{\mathrm{nav}}(v(x))}$). For dynamic scenes, 5–20 Hz receding-horizon replanning is implemented according to the VIO keyframe addition frequency.

**Figure 4:**
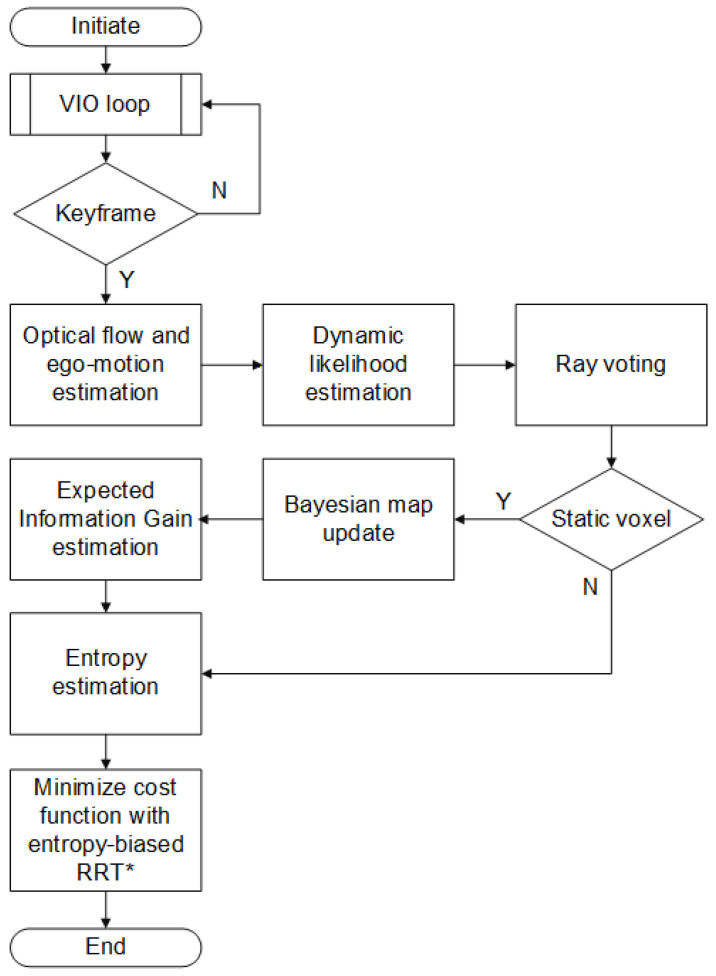
Flowchart of the proposed multi-criteria entropy-regularized path planning method.

The overall pipeline for multi-criteria path optimization with entropy regularization is summarized in Figure 4. The figure illustrates the sequential integration of perception, probabilistic mapping, information-gain evaluation, and entropy-biased planning within a receding-horizon control loop. Each stage transforms sensory observations and map priors into progressively refined navigation decisions, balancing exploration and safety in dynamic environments.

## 3. Results

To assess the effectiveness of the proposed entropy-regularized path-planning method, experiments evaluated mapping accuracy, computational efficiency, and robustness in dynamic environments. The evaluations were conducted both in simulation and on embedded hardware, specifically a Raspberry Pi 5 paired with a Hailo-8 AI Kit neural processing unit (NPU) capable of delivering up to 26 tera-operations per second (TOPS), as well as on a desktop-class system used as a performance reference. This experimental setup benchmarks not only the algorithm’s accuracy but also its suitability for deployment on resource-constrained platforms.

To validate the proposed method against precise ground truth, the NVIDIA Isaac Sim platform [31] was employed, providing photorealistic rendering and high-fidelity physics simulation. A digital twin of an industrial warehouse environment (20 × 20 m) was constructed, containing static obstacles (e.g., racks and machinery) as well as dynamic agents (e.g., forklifts and human workers) following randomized trajectories (speeds 0.5–2.0 m/s).

### 3.1. Accuracy of Entropy-Regularized Representation

The first set of experiments focused on validating the accuracy of the proposed entropy-based representation in distinguishing navigable from non-navigable regions. Table 2 compares the proposed approach against several baselines: (i) a standard occupancy grid without entropy regularization (Default); (ii) probabilistic occupancy mapping without explicit dynamic object modeling; and planning-based methods including the Dynamic Window Approach (DWA), Timed Elastic Band (TEB), and Model Predictive Path Integral (MPPI). For the latter three methods, mapping-related metrics reflect the performance of the underlying probabilistic baseline, while trajectory-related metrics capture how effectively each planner utilizes the available information for obstacle avoidance. All baseline planners were fine-tuned using grid search on a calibration dataset (five runs per method) to maximize success rate and mitigate hyperparameter bias.

Accuracy was evaluated using multiple complementary criteria. Static occupancy classification was assessed using the Intersection-over-Union (IoU) metric between predicted free/occupied regions and ground truth annotations. Dynamic object detection performance was quantified using the area under the receiver operating characteristic curve (AUC) for distinguishing moving from static entities. Trajectory accuracy was measured using the root mean squared error (RMSE) between planned paths and manually annotated ground truth safe trajectories. Finally, the trajectory safety rate is formally defined as the percentage of trials where the minimum Euclidean distance between the robot’s bounding box and any environmental obstacle remains above a strict safety threshold (0.1 m in our case) for the entire path.

The results indicate that entropy-regularized mapping consistently improves segmentation quality (static vs. dynamic), particularly in cluttered regions with partial occlusions. Crucially, these improvements in perception metrics (IoU and AUC) translate directly into enhanced navigation reliability. High IoU values ensure that narrow static passages are correctly identified, thereby reducing RMSE by preventing unnecessary detours. Meanwhile, improvements in AUC for dynamic classification correlate with a substantial increase in trajectory safety rate, rising from approximately 83% for probabilistic mapping to 92.7% for S3PM. In such environments, traditional occupancy grids tend to overestimate free space, while probabilistic baselines frequently misclassify ambiguous voxels. The proposed method mitigates these errors by weighting voxel states according to entropy, resulting in smoother and more reliable navigation maps.

**Figure 5:**
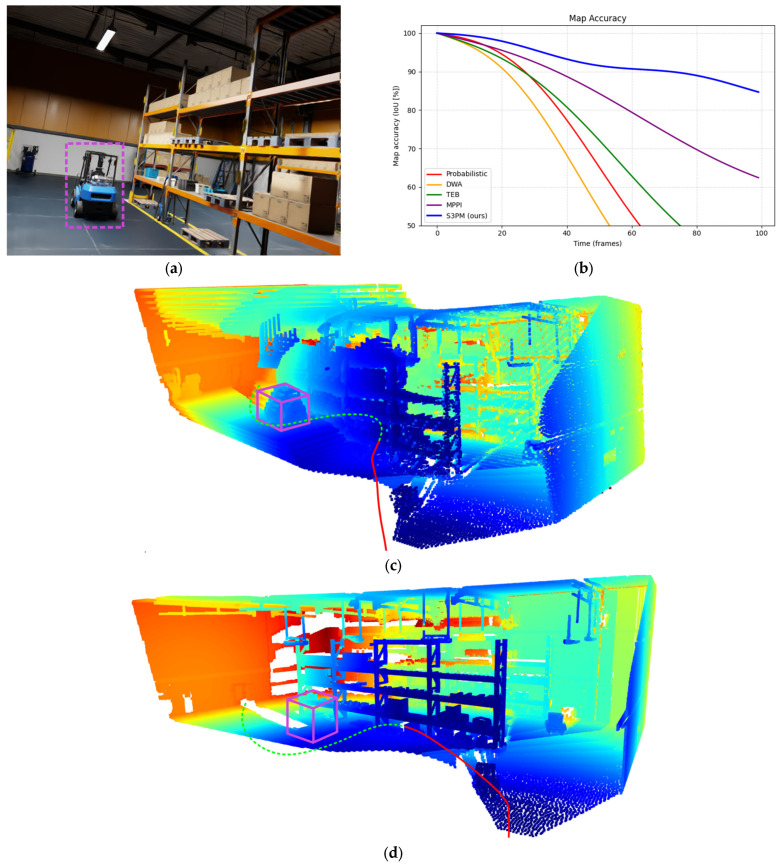
Qualitative evaluation of mapping accuracy and path planning in dynamic environments: (**a**) Original RGB image from the current camera frame with the marked forklift; (**b**) Comparison of reconstructed occupancy maps over the first 100 frames (probabilistic occupancy grid vs. the proposed S3PM method); (**c**) Point cloud and planned trajectory generated using probabilistic occupancy grid with RRT* planner; (**d**) Point cloud and planned trajectory generated using S3PM with entropy-regularized RRT*. Color legend: violet cube—dynamic object; dash green line—planned trajectory; solid red line—executed trajectory.

Representative results are shown in Figure 5, which presents qualitative comparisons of reconstructed 3D point clouds and the corresponding planned trajectories. The visualizations highlight the ability of the entropy-regularized formulation to preserve navigability and spatial consistency even in the presence of significant motion and occlusion.

**Figure 6:**
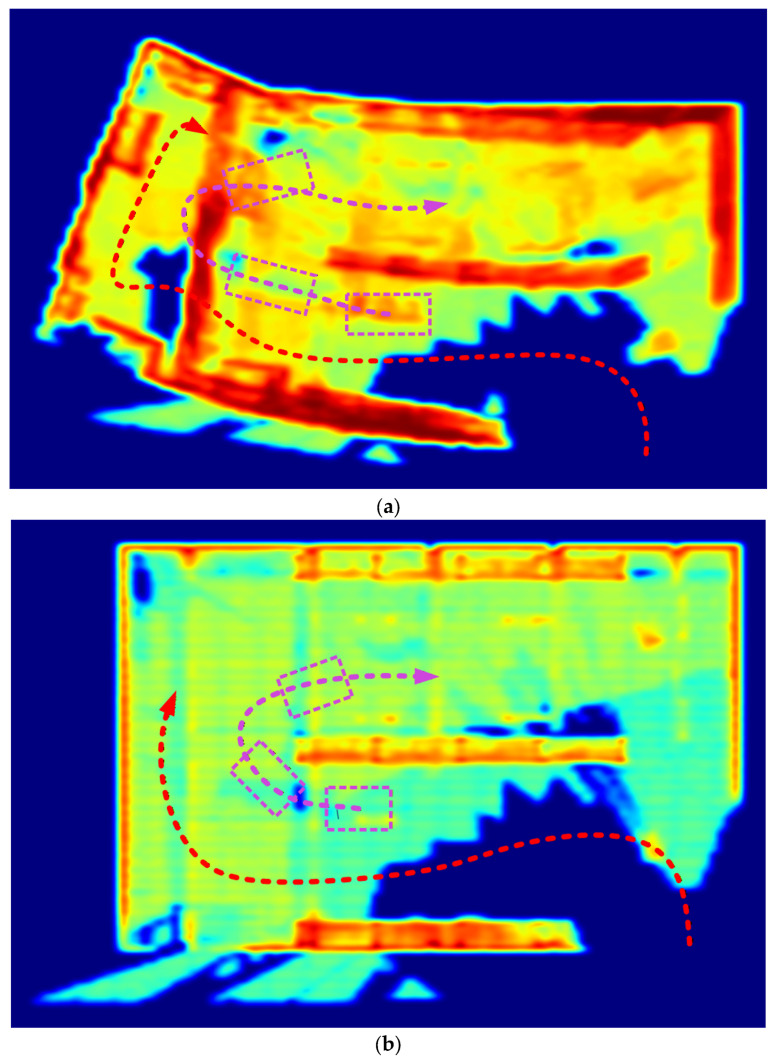
Top-down view of occupancy and trajectory distributions under dynamic conditions: (**a**) Top-down projection showing trajectory and occupancy distribution using probabilistic occupancy grid; (**b**) Top-down projection showing trajectory and occupancy distribution using S3PM with entropy-regularized mapping. Color legend: violet—dynamic object; red—executed trajectory.

To further assess the robustness of the proposed approach, additional experiments were conducted in environments containing moving agents and other transient obstacles. These trials demonstrate the algorithm’s ability to maintain stable path-planning performance under dynamic conditions, in which both scene structure and obstacle configurations evolve over time (Figure 6).

### 3.2. SP3M Efficiency

The second evaluation concerns runtime and memory performance. The experiments were conducted on three platforms:

1. Raspberry Pi 5 (Broadcom BCM2712 ARM Cortex-A76 CPU @ 2.4GHz, 8 GB LPDDR4X RAM, manufactured by Sony UK Technology);
2. Raspberry Pi 5 + Hailo-8 AI Kit (same as 1 + 26 TOPS NPU acceleration, manufactured by Hailo.ai);
3. Desktop reference system (Intel Core i7-12700K CPU @ 3.6GHz, 32 GB DDR5 RAM, NVIDIA RTX 3080 GPU).

We measured runtime of each method step (optical flow estimation, residual flow computation, entropy update, distance transform maintenance, and path planning) averaged over 100 planning cycles in indoor navigation scenarios (Table 3).

The results show that while the desktop-class Core i7 executes the full pipeline comfortably above 30 Hz, the Raspberry Pi 5 operating alone sustains approximately 12–15 Hz. This performance remains within acceptable real-time bounds for applications involving slow motion, such as inspection tasks in unstructured industrial environments. With the addition of the Hailo-8 AI Kit, computationally intensive stages—including optical flow estimation and residual computation—are offloaded, increasing the overall throughput to 25–30 Hz. Memory usage is efficiently bounded through voxel hashing, with typical consumption remaining below 1 GB for a rolling map window of 20 × 20 × 6 m.

## 4. Conclusions

This paper introduced S3PM, a lightweight entropy-regularized framework for simultaneous mapping and path planning in dense 3D point clouds within unstructured and dynamic industrial environments. The key contribution lies in the integration of a dynamic-aware entropy field that fuses voxel-wise occupancy probabilities with motion evidence extracted from residual optical flow. This representation explicitly quantifies both geometric uncertainty and object dynamics, enabling the system to distinguish reliable free space from ambiguous or high-risk regions in real time.

Extensive experiments conducted in simulation and on real hardware—including a Raspberry Pi 5 with and without Hailo-8 NPU acceleration—demonstrate clear advantages over established baselines in terms of navigation robustness, safety, and computational efficiency:

- An 18–27% higher IoU for static/dynamic segmentation;
- A 0.94–0.97 AUC for motion detection;
- A 30–45% reduction in collision events;
- Sustained real-time performance of 12–15 Hz on the bare Raspberry Pi 5 and 25–30 Hz with NPU offloading;
- Memory footprint below 1 GB for 20 × 20 × 6 m rolling maps.

These results confirm that entropy regularization, combined with incremental distance transforms and voxel hashing, significantly enhances navigation safety, localization robustness, and trajectory quality in cluttered environments with moving agents, while preserving the computational efficiency required for deployment on low-power embedded platforms.

Beyond its immediate industrial applicability, S3PM establishes a generalizable foundation for entropy-driven navigation strategies that can be extended to other high-uncertainty domains, such as search and rescue, planetary exploration, and service robotics in crowded public spaces. Future work will focus on integrating learned priors and multi-robot collaboration to further improve scalability and long-term autonomy.

## Figures and Tables

**Table 1:** Impact of cost function weights on pipeline performance.

$w_{d}$	$w_{\kappa }$	$w_{\mathrm{nav}\;}$	$w_{\mathrm{eig}\;}$	Path Length [m]	Minimum Obstacle Distance [m]	Newly Explored Voxels [Count (10^5^) per Keyframe]	* Path Smoothness [rad]
0.25	0.25	0.25	0.25	54.2	0.48	0.21	0.42
0.40	0.20	0.20	0.20	53.0	0.42	0.16	0.47
0.20	0.40	0.20	0.20	55.0	0.51	0.18	0.28
0.20	0.20	0.40	0.20	55.7	1.23	0.15	0.45
0.20	0.20	0.20	0.40	56.9	0.40	0.34	0.52
0.40	0.20	0.20	0.20	52.6	0.37	0.13	0.62
0.20	0.40	0.30	0.10	57.0	1.06	0.18	0.25
0.15	0.15	0.15	0.55	58.8	0.34	0.41	0.69
0.30	0.30	0.20	0.20	53.9	0.45	0.17	0.31

* Path smoothness is quantified as the average angular deviation between consecutive path segments, measured in radians. Lower values indicate smoother trajectories with fewer abrupt changes in direction.

**Table 2:** Performance comparison of the proposed S3PM and baseline methods. Values in parentheses represent the relative change compared to the “Default” baseline.

Method	Map Accuracy (IOU [%])	Map Awareness (AUC [%])	Trajectory Accuracy (RMSE [m])	Trajectory Safety Rate [%]
Default	71.40	76.20	0.54	52.30
Probabilistic	82.60 (+15.69%)	85.10 (+11.68%)	0.41 (−24.07%)	83.12 (+58.93%)
DWA	80.20 (+12.32%)	81.10 (+ 6.68%)	0.49 (− 9.26%)	78.20 (+49.52%)
TEB	83.40 (+16.81%)	86.20 (+13.12%)	0.35 (−35.18%)	88.50 (+69.22%)
MPPI	85.10 (+19.19%)	88.70 (+16.40%)	0.37 (−31.48%)	94.10 (+79.92%)
S3PM (ours)	89.80 (+25.77%)	92.40 (+21.26%)	0.38 (−29.63%)	92.70 (+77.25%)

**Table 3:** Computational performance breakdown of S3PM on embedded and desktop systems.

S3PM Step	Raspberry Pi 5	Raspberry Pi 5 + Hailo-8	Desktop
Optical Flow Estimation [ms]	22.5	6.8	4.1
Ego-motion Compensation [ms]	5.7	3.9	2.6
Dynamic Likelihood & Voting [ms]	18.2	7.4	5.3
Entropy Update & Regularization [ms]	14.6	6.2	4.8
Distance Transform [ms]	9.8	5.3	3.1
Path Planning [ms]	11.4	5.9	4.2
Average Runtime per Cycle [ms]	82.2	35.5	24.1
Average Frequency [Hz]	12.2	28.1	41.5
Idle Power [W] (System only)	3.1	3.4	45.2
Average Power Consumption (S3PM) [W]	6.8	8.2	127.9
Peak Power Consumption (S3PM) [W]	9.4	11.6	210.5

## Data Availability

Data available on request due to restrictions.
